# Ticagrelor-induced Diarrhea in a Patient with Acute Coronary Syndrome Requiring Percutaneous Coronary Artery Intervention

**DOI:** 10.7759/cureus.3874

**Published:** 2019-01-12

**Authors:** Mohammad Alomari, Hunter Bratton, Ahmad Musmar, Laith A Al Momani, Mark Young

**Affiliations:** 1 Internal Medicine, Fairview Hospital, Cleveland, USA; 2 Internal Medicine, East Tennessee State University, Johnson City, USA; 3 Internal Medicine, Florida Atlantic University, Pompano Beach, USA

**Keywords:** ticagrelor, diarrhea, p2y12 inhibitor, adverse effects, acute coronary syndrome, percutaneous coronary artery intervention

## Abstract

The P2Y_12_ inhibitor, ticagrelor, has been shown to prevent thrombotic events and hence, improve morbidity and mortality in patients with acute coronary syndrome following coronary artery stent placement. Despite many clinical benefits, ticagrelor has been associated with several adverse effects, including dyspnea, easy bruising, and gastrointestinal bleeding. We report the case of a 67-year-old patient with an acute coronary artery syndrome requiring percutaneous coronary artery intervention with stenting who developed ticagrelor-induced diarrhea. The patient’s ticagrelor medication was replaced with clopidogrel, and his diarrhea completely resolved within one week with no complications observed at his one-month follow-up visit. Clinicians should be aware of this adverse effect of ticagrelor so as to guide them toward possible underlying etiologies and appropriate workup of chronic diarrhea.

## Introduction

Cardiovascular disease causes more than 600,000 deaths in the United States annually and composes 17% of all health expenditures [1-2]. In 2011, the Food and Drug Administration approved ticagrelor, a P2Y_12_ inhibitor, to prevent thrombotic events and improve the care of patients with acute coronary syndrome. Despite its clinical benefits, ticagrelor has been associated with many adverse effects [3-4]. Dyspnea, bleeding, and easy bruising are the most commonly reported side effects, while gastrointestinal bleeding is one of the most worrisome complications [5-8]. In this report, we present the case of a 67-year-old patient with a history of acute coronary syndrome requiring percutaneous coronary artery intervention. To the best of our knowledge, this case is the first report of severe diarrhea attributed to ticagrelor that necessitated premature discontinuation of the medication.

## Case presentation

A 67-year-old man presented to the clinic for follow-up of celiac disease and microscopic colitis diagnosed five years prior. He complained of six to seven large, loose bowel movements daily starting 10 months prior to this visit. His symptoms began soon after a prior admission for acute coronary syndrome requiring percutaneous coronary artery intervention. Review of systems was negative for dark stools, hematochezia, and abdominal pain. He denied any upper gastrointestinal symptoms, and he was adherent to a strict gluten-free diet. His last upper and lower endoscopies were five years prior to presentation, both of which were normal examinations. Biopsies were consistent with celiac disease and microscopic (lymphocytic) colitis.

The patient was started on aspirin and ticagrelor after his coronary artery stent placement 10 months prior. Additionally, he was on lisinopril and atorvastatin.

Physical examination was unremarkable. Laboratory workup, including a complete blood count (CBC) and comprehensive metabolic panel (CMP), was also unremarkable. Further testing revealed a negative Clostridium difficile polymerase chain reaction (PCR), negative stool studies for Giardia, and a negative enzyme-linked immunosorbent assay (ELISA) test for immunoglobulin A (IgA) tissue transglutaminase antibodies. The patient continued to have diarrhea despite multiple trials of different interventions, including budesonide, cholestyramine, atropine/diphenoxylate, and bismuth. Subsequently, a course of rifaximin for possible small intestinal bacterial overgrowth was tried, which yielded no change in symptoms.

The possibility of ticagrelor being the offending agent was considered, as the onset of diarrhea corresponded with the time of initiation of treatment. The patient’s ticagrelor medication was replaced with clopidogrel, and his diarrhea completely resolved within one week. The patient had no complaints at his one-month follow-up visit, and he reported one to two well-formed stools per day.

## Discussion

Numerous studies, including the PEGASUS-TIMI 54 (Prevention of Cardiovascular Events in Patients With Prior Heart Attack Using Ticagrelor Compared to Placebo on a Background of Aspirin - Thrombolysis in Myocardial Infarction 54) trial, have recently demonstrated the benefit of extended dual antiplatelet therapy beyond one year in patients with prior myocardial infarction [9-11]. Additionally, several studies have demonstrated the cost-effectiveness of dual antiplatelet therapy, suggesting a larger percentage of patients will likely find themselves taking ticagrelor for longer periods of time following cardiac events, putting them at an increased risk for adverse events [12-15].

In the clinical setting, premature ticagrelor cessation occurs in one out of six patients. These patients often cite medication adverse effects as the reason behind the discontinuation, with bleeding and dyspnea being the most frequently endorsed [8]. Furthermore, adverse effects are the most common reason to switch to a different P2Y_12_ therapy [8]. In 2013, Serebruany et al. used two large acute coronary syndrome trials, PLATO (PLATelet inhibition and patient Outcomes trial; ticagrelor versus clopidogrel in patients with acute coronary syndromes) and TRITON (TRial to assess Improvement in Therapeutic Outcomes by optimizing platelet inhibitioN; prasugrel versus clopidogrel in patients with acute coronary syndromes), to match the risks of gastrointestinal complications among antiplatelet regimens. Their data demonstrated that ticagrelor has a 1.12 relative risk compared to clopidogrel of causing diarrhea with 4.14% of patients reporting diarrhea as an adverse event [16]. However, neither of these studies commented on any resultant drug discontinuation.

In May 2018, research by Zanchin et al. demonstrated bleeding (41%), dyspnea (29%), and gastrointestinal symptoms (18%) were responsible for 88% of adverse effects leading to premature ticagrelor cessation [8]. However, it is not evident from Zanchin et al. whether diarrhea was included as a gastrointestinal symptom, and if so, what percentage of the patients who stopped taking ticagrelor for gastrointestinal symptoms did so because they endorsed diarrhea. A literature review found no other study documenting diarrhea as an adverse effect of ticagrelor; to our knowledge, this is the first case report to demonstrate diarrhea as a side effect by showing alleviation of the patient’s diarrhea through discontinuation of the medication.

Numerous mechanisms have been explored as causes of nausea and diarrhea, including afferent vagal neurons of the intestinal mucosa being stimulated by serotonin (5-hydroxytryptamine, (5-HT)) released from endocrine cells [17]. Nausea and diarrhea can also result from direct binding of drugs, toxins, and neurotransmitters (such as 5-HT) to receptors within the gastrointestinal tract [18]. Previous studies of ticlopidine, clopidogrel, and prasugrel have hypothesized that the gastrointestinal adverse events caused by these medications are the result of direct activation of the Ca2+-permeable ion channel transient receptor potential ankyrin 1 (TRPA1) expressed in the small intestine, which causes secretion of 5-HT from enterochromaffin cells [19]. A 2013 study by Berger proposed that off-target gastrointestinal effects should be considered for pharmaceuticals that irreversibly bind P2Y_12_ receptors [20]. In 2016, Lian-Rico et al. suggested that drugs that target the P2Y_12_-adenyl cyclase (AC)/cyclic adenosine monophosphate (cAMP) signaling pathway can contribute to improper mechanosensation of enterochromaffin cells causing abnormal gut reflexes and symptoms of diarrhea, constipation, and visceral pain.

Given the number of patients who are candidates for ticagrelor therapy following a cardiac event, it is critically important to increase awareness of ticagrelor-induced diarrhea among clinicians, identify accurate rates of this adverse event, and determine the mechanism behind this undesired effect. Further investigation of the ability of ticagrelor to stimulate 5-HT release from enterochromaffin cells is warranted as this mechanism has been identified in other P2Y_12_ inhibitors and may explain this patient’s ticagrelor-induced diarrhea.

## Conclusions

In conclusion, a literature review shows a very limited number of studies documenting ticagrelor-associated diarrhea. To our knowledge, no previous case reports have discussed the severity of symptoms, especially to the extent of drug discontinuation. Increased awareness of such adverse effects and a thorough medical history can guide clinicians toward possible underlying etiologies and appropriate workup of chronic diarrhea.
